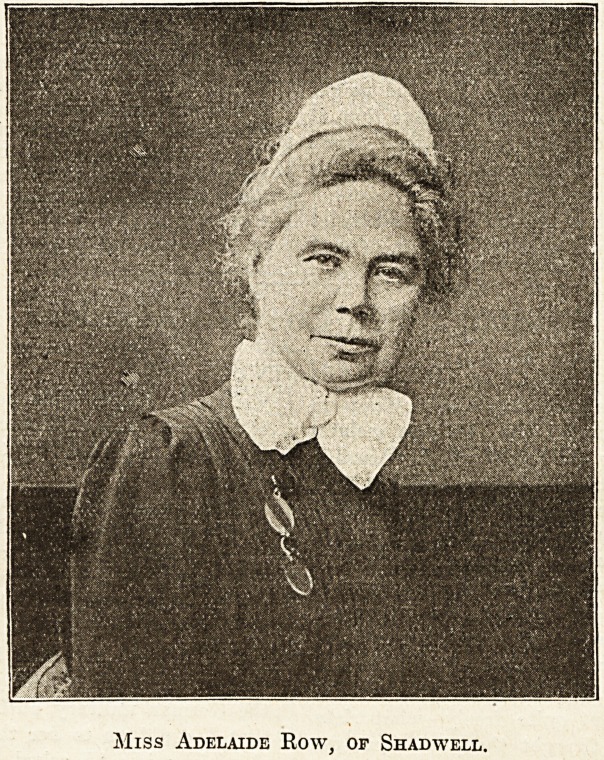# A Pioneer Matron

**Published:** 1918-12-28

**Authors:** 


### A PIONEER MATRON.

### Thirty-five Years' Hospital Work.

The retirement of Miss Row from the office of matron at the East London Hospital for Children, Shadwell, which she had held for nearly a quarter of a century, is one of the events which have some historic importance from the nursing point of view. One by one they are leaving their well-loved work, these pioneer matrons, and so little is the story of nursing understood that even now many will be found to inquire "Why are they called Pioneers?"

The matrons on whom this title may be conferred are the first who, being themselves nurses, assumed, in any institution for the sick, the superintendence of the nursing staff. We have no call to find fault with the women who preceded them. Some are still alive, and very many performed their duties with devotion and in singleness of heart. But the gulf which lies between a matron who is a nurse and a matron who is a lay woman can be appreciated by all. The first trained nurse who held sway in her hospital as matron was of necessity a pioneer.

Miss Row's probationer days go back to the year 1883, and it was in the hospital she has ruled for so many years that she first started. Not many educated women entered the nursing profession in those days, and few indeed of these went down to the East End for training. Yielding to the wishes of her friends Miss Row entered for a year as lady probationer at a payment of a guinea a week, and was rewarded with the boon of a room to herself. Other probationers slept four in a room, without screens, sharing washstand, chest of drawers, and dressing-table in pairs. The fare was plentiful and the diet largely of meat. The night nurses, for instance, had for their breakfast before going on duty the same fare as the day nurses for supper—cold meat, bread and cheese, and beer. The tea for which they craved had to be made later in their wards. It was a hard life but not unhealthy. They were always short-handed. For the ninety-one beds there was a nominal staff of twenty-five, but it seldom numbered more than twenty or twenty-one. The hours were long, and the night nurses were on duty from 9 P.M. till 10 A.M. Off-duty time came when it could be fitted in, about 2½ hours twice a week, often omitted. No whole day was ever given, for always the morning work up to 10 A.M. had to be finished before departure. Lectures came very spasmodically; when some doctor could be induced to oblige, and there were no examinations. The main thing to learn was the system of dressing wounds under the carbolic spray, a matter of some delicacy, then in vogue in all institutions for the sick. Miss Row looks back with some amusement now to the thought of those early days when she went about her duties attired in a black alpaca dress which touched the ground, the cotton frock assumed for dirty work in the early morning being discarded at 10 A.M., when she dressed for the day. The ordinary probationers wore similar long gowns of grey linsey.

Miss Adelaide Row, of Shadwell.

After a year at the East London Hospital Miss Row went to St. Bartholomew's, where Mrs. Bedford Fenwick was in the midst of her short but brilliant career as matron. Here there was already the nucleus of a modern training-school, regular lectures and examinations. After two months' probation Miss Row became staff-nurse in Harley Ward. Her hours were easier and the work exceedingly interesting. The spray dressings were still used in some of the wards, while in others they were replaced by antiseptic lotions. Aseptic treatment came in slowly and at first experimentally. Off-duty times were from two to three hours twice a week, with part of Sunday, and a day starting at 10 A.M., once a fortnight. The older nurses still wore their stuff dresses, and conditions differed radically from those of the present day. Miss Row returned to Shadwell as out-patient sister on leaving St. Bartholomew's, and held that post for one year, after which for seven years she was sister of Enfield Ward. In 1894 she was appointed matron of the Jenny Lind Hospital at Norwich, a post which she held for one year only, for she returned in 1895 at the call of those who knew her past work to become matron of the East London Hospital for Children.

During the twenty-three years she passed in this institution, it underwent that slow transformation which succeeded at last in establishing its reputation as second to none in its own branch of work. The number of beds rose to 130, and the staff was doubled, while, needless to say, the call is still for more room. The nurses' quarters have been completely overhauled, and a fresh block of well-designed cubicles has relieved their over-crowded quarters. Many of the staff have good separate rooms. A comfortable sick-room has also been added. An isolation block, indispensable in dealing with young children, together with a good steam laundry, have been built on, and in 1908 a well-designed system of balconies and shelters enabled open-air treatment to be further developed.

**A Pioneer Matron**—(*continued*).

The passage of years has seen many curious changes in nursing, but none is more far-reaching in its effects than that which converted open air from being regarded as the relentless enemy of little children to its position as their saviour and best friend. It used, of course, to be the rule to exclude every breath of outside air from pneumonia patients, and the wards in which these little sufferers were carefully tended were kept at a high temperature, with closed windows night and day. The result was a high mortality from this complaint. One of the sisters noticed from her own observation that cases which were for any cause less rigidly excluded from a free current of air did better than the others and began cautiously to experiment on her own account with the matron's consent. It became almost instantly evident that the admission of air relieved the breathing and exercised a remarkable effect in soothing the sufferers. More than that, it began to be clear that the babies took pneumonia because they were enfeebled, and that in airy wards the dreaded malady kept aloof. Thus some time before the official edict went forth, the open-air treatment had its votaries at the East London Hospital for Children. It is now one of the most powerful centres of fresh-air propaganda in the East End. For the neighbourhood, though still very poor, is less ignorant than it used to be, and the mothers, many of whom were patients in old days, are apt to learn and improve their own environment.

The training-school is now first rate and able to attract always, except during the last few busy months, a good supply of educated probationers. It has passed into very able hands in the person of Miss Coulton. The rise in value of the posts it offers may be gauged from the fact that in old days the sisters received £25 a year, out of which they paid for their own washing. This recurrent drain on their slender income became at last intolerable, and, growing suddenly articulate, the sisters sent a deputation to the board-room to ask for washing money, and were gratified by an immediate increase of £5 a year, a sum which was not deducted when, after a short period, they were permitted the privilege of free laundry.

Through all the crowded detail of a life lived in a singularly hidden routine, it is easy to see that the pioneer matron has kept clear the ideals of her youth. Every year has taught something which the nurse in training needs if she is to be entrusted with that most elusive being, a sick baby. More and more the hospital serves the infant population, some two-thirds of the beds being usually occupied by infants under two years, and here, unless there be understanding and that intuitive sympathy which is like a sixth sense, life will often quietly leave the little body, like a candle extinguished by a puff of air. A passing shadow, the merest surface alteration, is often the only symptom the infant can give of an oppression which means death unless relieved. To ensure that eye and hand are trained to an exquisite pitch of skill, in which the heart surely takes its part, is work which requires a very able body of nursing teachers. To organise and perfect such a centre of nursing education requires the undivided allegiance of an able woman gifted in no ordinary degree with similar skill. To have succeeded as Miss Row has succeeded is a crown of rare achievement.

## Figures and Tables

**Figure f1:**